# Supplementary material for: Who gets sicker and why? Parents’ perceptions of COVID-19 disparities and how they would explain them to their children
Source: PLoS One. 2025 Oct 15;20(10):e0332140. doi: 10.1371/journal.pone.0332140 (PMC12527206; doi:10.1371/journal.pone.0332140)
Supplement: S1 File — (DOCX) [file pone.0332140.s001.docx]

**Figure S1: Proportion of disparity judgment scale task responses by disparity judgment (forced-choice) response per comparison**

**
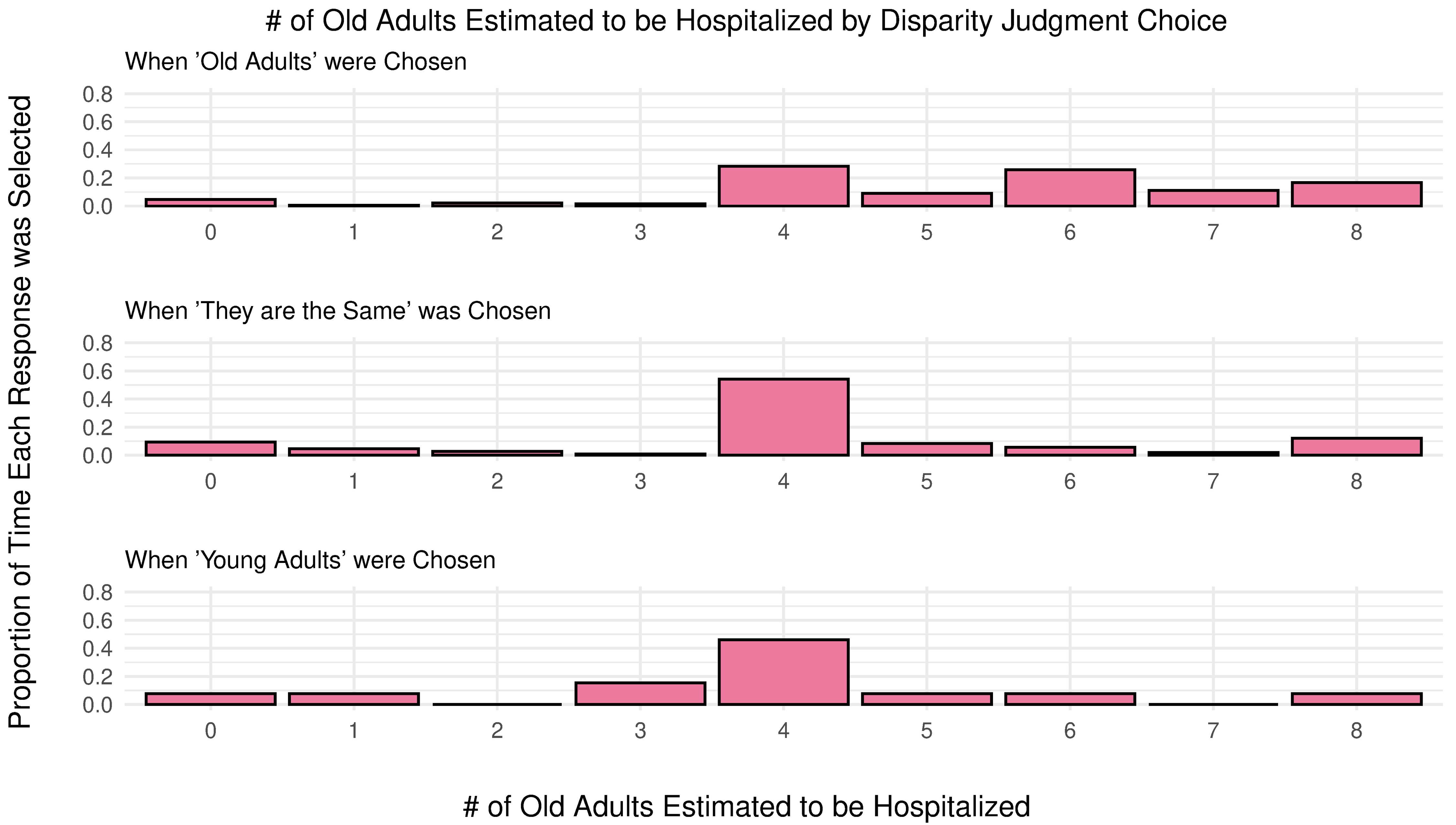
**

**
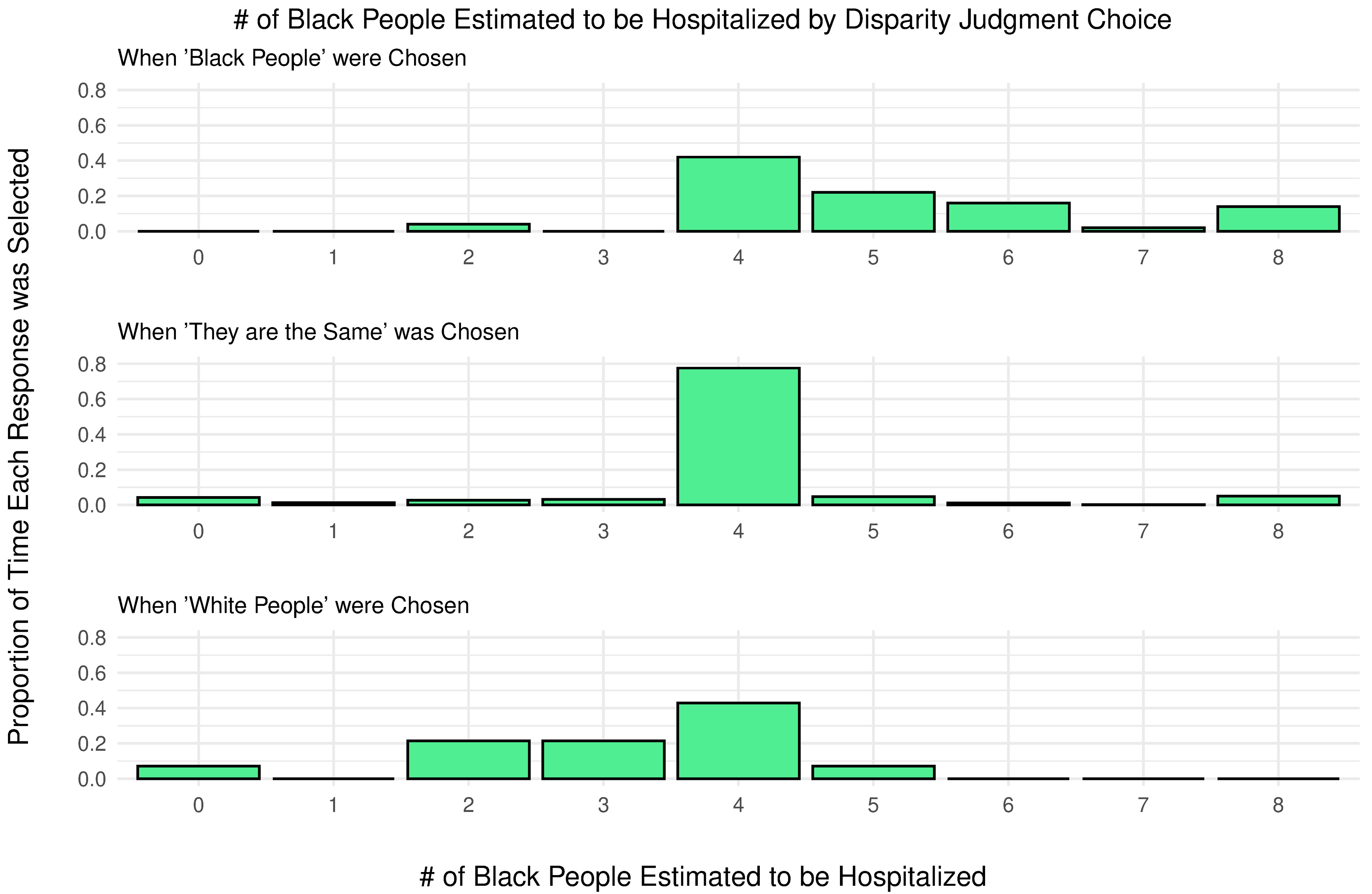
**


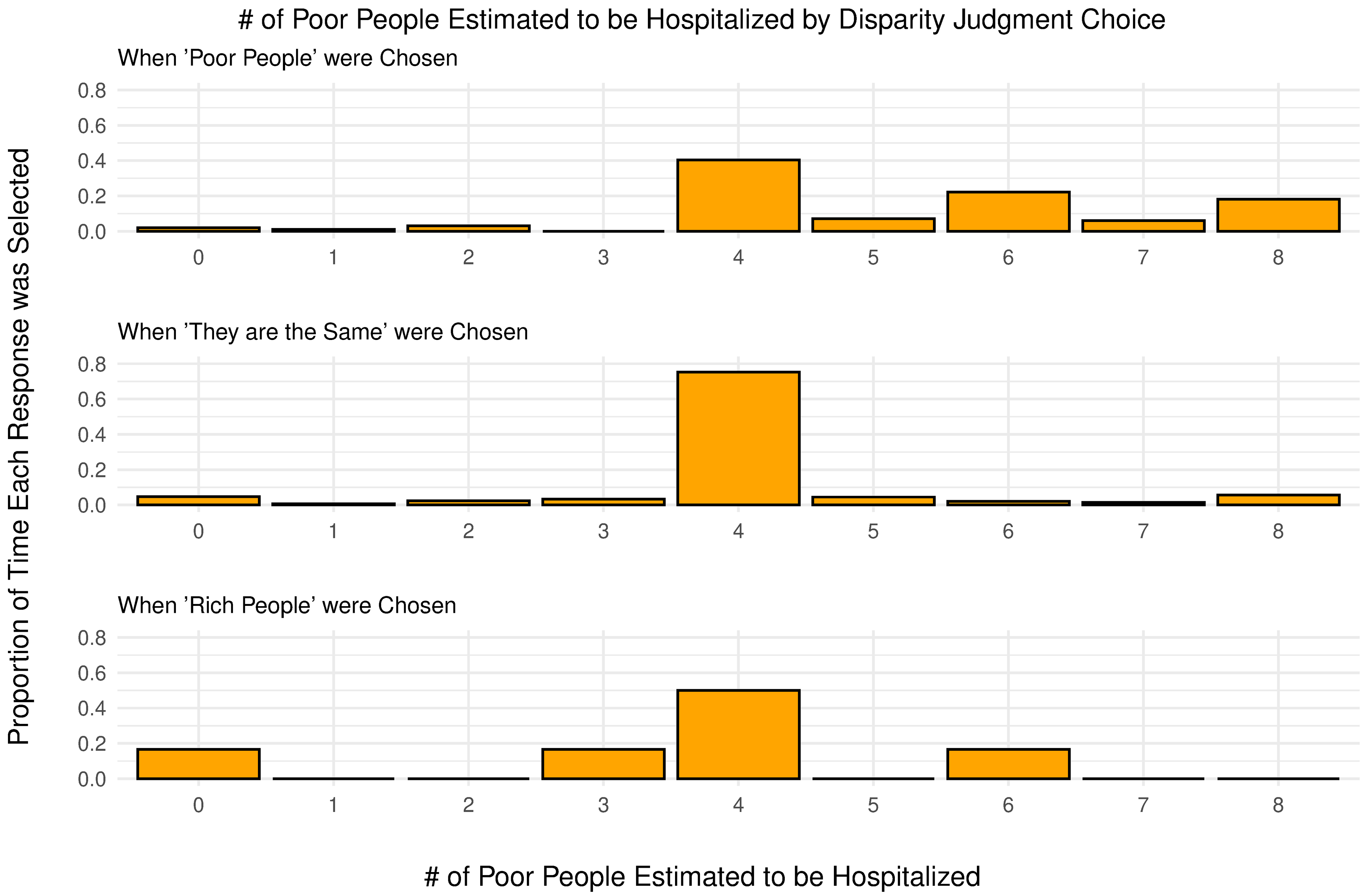


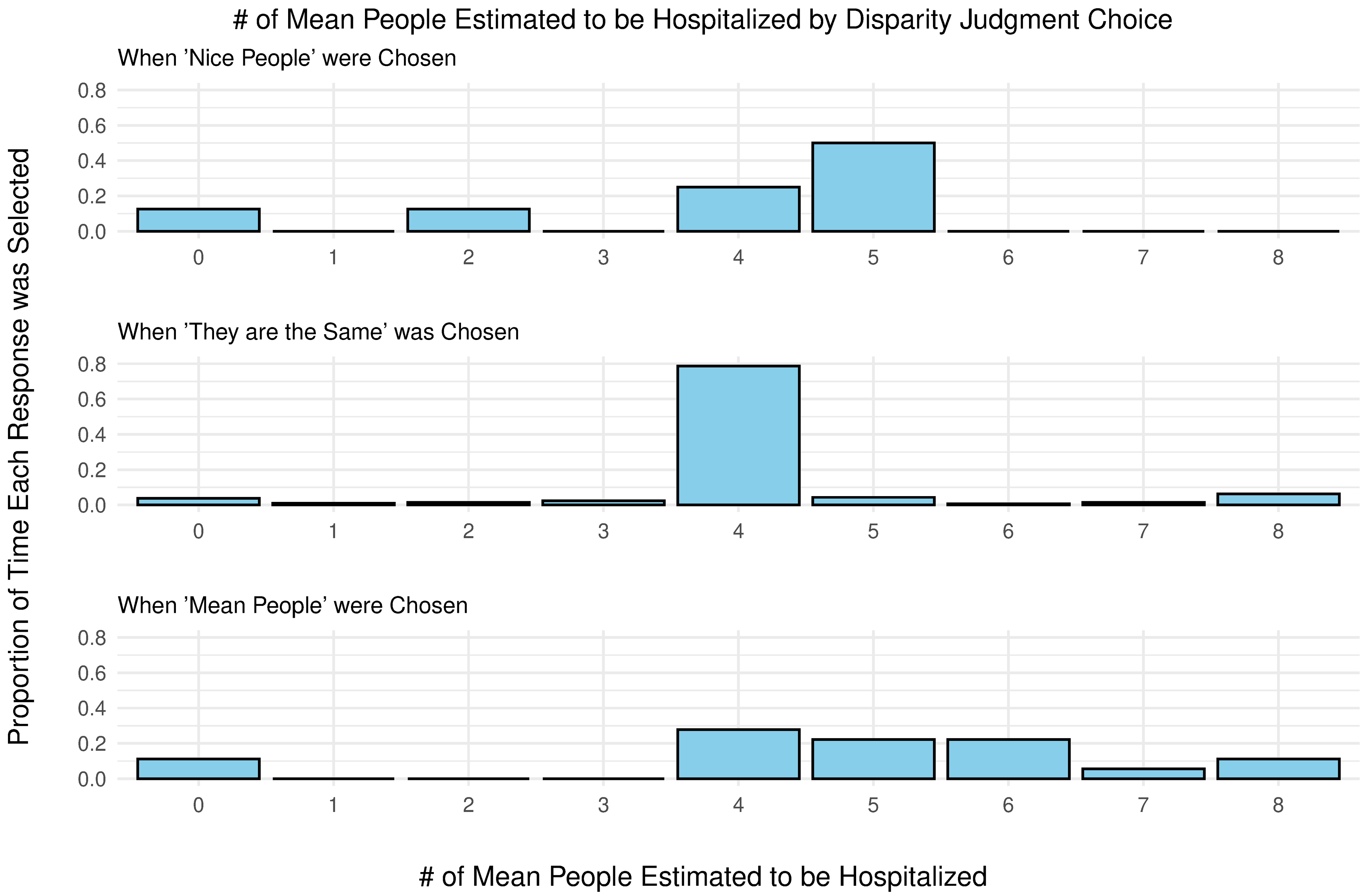


**Explanation endorsement by disparity judgment**

We fit mixed-effects logistic regressions predicting whether parents said “yes” to a given explanation, with explanation type (behavioral, biological, structural) as a fixed effect, and participant as a random effect. We conducted two regression analyses per comparison, one analyzing the endorsements of those parents who selected one of the two groups as more likely to get sick, and the second analyzing the endorsements of those parents who selected “they are the same”. When an effect of explanation type was present, we conducted pairwise comparisons to determine which endorsements were significantly different using odds ratios (OR) using the *emmeans* package in R. Asymptotic confidence intervals and *p*-values were adjusted using the Tukey method for comparing a family of 3 comparisons per analysis (see Tables S1 and S2).

**Age (Old vs. Young)*.*** For parents who chose one of the two age groups in the Disparity Judgment (forced-choice) task, there was a significant effect of explanation type (χ^2^(2, *n* = 335) = 155.22, *p* < .001). They endorsed the biological explanation more than the behavioral (*p* < .001) or structural (*p* < .001) explanations and endorsed the behavioral explanation more than the structural (*p* = .008) explanation. For parents who chose both groups as equally likely to get sick in the Disparity Judgment (forced-choice) task, there was a significant effect of explanation type (χ^2^(2, *n* = 107) = 16.98, *p* = .002). Parents endorsed the biological explanation more than the behavioral (*p* = .008) or structural (*p* = .002) explanations. There was no significant difference between the behavioral and the structural explanations (*p* = .43).

**Race (Black vs. White).** For the parents who chose one of the two racial groups in the Disparity Judgment (forced-choice) task, there was a significant effect of explanation type (χ^2^(2, *n* = 64) = 10.84, *p* = .004). They endorsed the biological explanation more than the behavioral explanation (*p* = .03) and the structural explanation more than the behavioral explanation (*p* = .005). There was no significant difference between the biological and structural explanations (*p* = .849). For the parents who chose that both racial groups were equally likely to get sick in the Disparity Judgment (forced-choice) task, there was a significant effect of explanation type (χ^2^(2, *n* = 378) = 125.31, *p* < .001). They endorsed the biological explanation more than the behavioral (*p* < .001) and structural (*p* < .001) explanations. There was no significant difference between the behavioral and structural explanations (*p* = .13).

**Social Class (Poor vs. Rich).** For the parents who chose one of the two social class groups in the Disparity Judgment (forced-choice) task, there was a significant effect of explanation type (χ^2^(2, *n* = 315) = 36.47, *p* < .001). They endorsed the structural explanation more than the behavioral explanation (*p* < .001). They endorsed the structural explanation more than the behavioral (*p* < .001) and biological (*p* < .001) explanations. There was no significant difference between the behavioral and the biological explanations (*p* < .465). For the parents who chose that both social class groups were equally likely to get sick in the Disparity Judgment (forced-choice) task, there was a significant effect of explanation type (χ^2^(2, n = 336) = 107.12, *p* < .001). They endorsed the biological explanation more than the behavioral (*p* < .001) and structural (*p* < .001) explanations. They endorsed the behavioral explanation more than the structural explanation (*p* = .003).

**Table S1. Odds ratio of endorsement (yes/no) by explanation type when participants chose that both groups were equally likely to get sick on the disparity judgment (forced-choice) task**

| **Pairwise Comparisons** | **OR** | **SE** | **95% Asymptotic OR CIs** | ***z*** |
| --- | --- | --- | --- | --- |
| **Age** |  |  |  |  |
| Behavioral – Biological | 0.33 | 0.12 | [0.14, 0.79] | - 3.00** |
| Behavioral – Structural | 1.56 | 0.56 | [0.672, 3.62] | 1.24 |
| Biological – Structural | 4.70 | 1.82 | [1.90, 11.64] | 4.01** |
| **Race** |  |  |  |  |
| Behavioral – Biological | 0.10 | 0.02 | [0.06, 0.17] | -9.67*** |
| Behavioral – Structural | 1.49 | 0.31 | [0.92, 2.43] | 1.92 |
| Biological – Structural | 15.21 | 3.91 | [8.32, 27.80] | 10.58*** |
| **Social Class** |  |  |  |  |
| Behavioral – Biological | 0.14 | 0.03 | [0.07, 0.24] | -8.28*** |
| Behavioral – Structural | 2.08 | 0.46 | [1.23, 3.49] | 3.30** |
| Biological – Structural | 15.28 | 4.15 | [8.09, 28.87] | 10.5*** |

*Note:* Asymptotic confidence intervals and *p*-values were adjusted using the Tukey method for comparing a family of 3 comparisons per analysis. * *p* < .05, ** *p* < .01, *** *p* < .001

**Table S2. Odds ratio of endorsement (yes/no) by explanation type when participants chose that one of the groups was more likely to get sick on the disparity judgment (forced-choice) task**

| **Pairwise Comparisons** | **OR** | **SE** | **95% Asymptotic OR CIs** | ***z*** |
| --- | --- | --- | --- | --- |
| **Age** |  |  |  |  |
| Behavioral – Biological | 0.02 | 0.01 | [0.01, 0.05] | - 11.50*** |
| Behavioral – Structural | 1.86 | 0.39 | [1.14, 3.04] | 2.985** |
| Biological – Structural | 80.08 | 28.83 | [33.44, 186.22] | 12.17*** |
| **Race** |  |  |  |  |
| Behavioral – Biological | 0.38 | 0.14 | [0.16, 0.91] | -2.60* |
| Behavioral – Structural | 0.31 | 0.12 | [0.13, 0.76] | -3.08** |
| Biological – Structural | 0.82 | 0.30 | [0.35, 1.93] | -0.55 |
| **Social Class** |  |  |  |  |
| Behavioral – Biological | 1.42 | 0.43 | [0.71, 2.88] | 1.18 |
| Behavioral – Structural | 0.09 | 0.04 | [0.04, 0.27] | -5.29*** |
| Biological – Structural | 0.06 | 0.03 | [0.02, 0.20] | -5.94*** |

*Note:* Asymptotic confidence intervals and *p*-values were adjusted using the Tukey method for comparing a family of 3 comparisons per analysis. * *p* < .05, ** *p* < .01, *** *p* < .001

**Table S3a. 𝜒2LR test predicting if discussion likelihood differs by child age and disparity type**

| **Predictors** | **𝜒2LR** | **df** | ***p*** |
| --- | --- | --- | --- |
| Child Age | 171.767 | 1 | < .001 |
| Disparity Type | 48.835 | 2 | < .001 |

**Table S3b. Discussion likelihood by child age and disparity type**

| **Predictors** | **OR** | **SE** | **95% Asymptotic OR CIs** | ***z*** |
| --- | --- | --- | --- | --- |
| Child Age | 1.16 | 0.05 | [1.06, 1.27] | 3.08** |
| Age v. Race | 2.51 | 0.14 | [1.92 – 3.29] | 6.67*** |
| Social Class v. Race | 1.28 | 0.13 | [0.98 – 1.65] | 1.84 |
| Age v. Social Class | 1.97 | 0.14 | [1.51 – 2.57] | 5.01*** |

*Note*: ** p* < .05, ** *p* < .01, *** *p* < .001.

**Table S4. Disparity judgment confidence rating task regression results for the age comparison**

| **Predictor** | **β** | **SE** | **95%CI** | ***t*** |
| --- | --- | --- | --- | --- |
| Intercept | 3.12 | 0.08 | [2.96, 3.28] | 38.31*** |
| Old or Young Adults | 0.02 | 0.09 | [0.16, 0.21] | 0.26 |

*Note*: ** p* < .05, ** *p* < .01, *** *p* < .001.

**Table S5. Disparity judgment confidence rating task regression results for the racial comparison**

| **Predictor** | **β** | **SE** | **95%CI** | ***t*** |
| --- | --- | --- | --- | --- |
| Intercept | 3.38 | 0.09 | [3.21, 3.56] | 37.41*** |
| Black or White People | -0.28 | 0.10 | [-0.49, -0.08] | -2.72** |

*Note*: ** p* < .05, ** *p* < .01, *** *p* < .001.

**Table S6. Disparity judgment confidence rating task regression results for the social class comparison**

| **Predictor** | **β** | **SE** | **95%CI** | ***t*** |
| --- | --- | --- | --- | --- |
| Intercept | 3.30 | 0.05 | [3.21, 3.39] | 71.77*** |
| Poor or Rich People | -0.22 | 0.09 | [-0.40, -0.03] | -2.29* |

*Note*: ** p* < .05, ** *p* < .01, *** *p* < .001.

**Table S7. Disparity judgment confidence rating task regression results for the personality comparison**

| **Predictor** | **β** | **SE** | **95%CI** | ***t*** |
| --- | --- | --- | --- | --- |
| Intercept | 3.35 | 0.04 | [3.44, 3.59] | 94.69*** |
| Mean or Nice people | -0.55 | 0.15 | [-0.85, -0.25] | -3.60*** |

*Note*: ** p* < .05, ** *p* < .01, *** *p* < .001.

**Table S8a. Explanation endorsement task Wald 𝜒2 test when participants chose that both groups were equally likely to get sick on the disparity judgment (forced-choice) task for the age comparison**

| **Predictor** | **𝜒2** | **df** | ***p*** |
| --- | --- | --- | --- |
| Explanation Type | 16.69 | 2 | < .001 |

*Note:* See Table S1 for pairwise comparisons.

**Table S8b. Explanation endorsement task Wald 𝜒2 test when participants chose that one of the groups was more likely to get sick on the disparity judgment (forced-choice) task for the age comparison**

| **Predictor** | **𝜒2** | **df** | ***p*** |
| --- | --- | --- | --- |
| Explanation Type | 155.22 | 2 | < .001 |

*Note:* See Table S2 for pairwise comparisons.

**Table S9a. Explanation endorsement task Wald 𝜒2 test when participants chose that both groups were equally likely to get sick on the disparity judgment (forced-choice) task for the race comparison**

| **Predictor** | **𝜒2** | **df** | ***p*** |
| --- | --- | --- | --- |
| Explanation Type | 125.31 | 2 | < .001 |

*Note:* See Table S1 for pairwise comparisons.

**Table S9b. Explanation endorsement task Wald 𝜒2 test when participants chose that one of the groups was more likely to get sick on the disparity judgment (forced-choice) task for the race comparison**

| **Predictor** | **𝜒2** | **df** | ***p*** |
| --- | --- | --- | --- |
| Explanation Type | 10.84 | 2 | < .001 |

*Note:* See Table S2 for pairwise comparisons.

**Table S10a. Explanation endorsement task Wald 𝜒2 test when participants chose that both groups were equally likely to get sick on the disparity judgment (forced-choice) task for the social class comparison**

| **Predictor** | **𝜒2** | **df** | ***p*** |
| --- | --- | --- | --- |
| Explanation Type | 107.12 | 2 | < .001 |

*Note:* See Table S1 for pairwise comparisons.

**Table S10b. Explanation endorsement task Wald 𝜒2 test when participants chose that one of the groups was more likely to get sick on the disparity judgment (forced-choice) task for the social class comparison**

| **Predictor** | **𝜒2** | **df** | ***p*** |
| --- | --- | --- | --- |
| Explanation Type | 36.47 | 2 | < .001 |

*Note:* See Table S2 for pairwise comparisons.

**Table S11a. Explanation endorsement task Wald 𝜒2 test when participants chose that both groups were equally likely to get sick on the disparity judgment (forced-choice) task for the personality comparison**

| **Predictor** | **𝜒2** | **df** | ***p*** |
| --- | --- | --- | --- |
| Explanation Type | 142.29 | 2 | < .001 |

*Note:* See Table S1 for pairwise comparisons.

**Table S11b. Explanation endorsement task 𝜒2 test when participants chose that one of the groups was more likely to get sick on the disparity judgment (forced-choice) task for the personality comparison**

| **Predictor** | **𝜒2** | **df** | ***p*** |
| --- | --- | --- | --- |
| Explanation Type | 2.40 | 2 | .30 |

*Note:* See Table S2 for pairwise comparisons.

**Table S12. Discussion likelihood as a function of beliefs and attitudes toward age groups**

| **Predictors** | **OR** | **SE** | **95%CI** | ***z*** |
| --- | --- | --- | --- | --- |
| Age Essentialism | 1.14 | 0.11 | [0.93 – 1.41] | 1.25 |
| Age Social Constructionism | 1.13 | 0.12 | [0.89 – 1.41] | 1.01 |
| Age Category Blindness | 0.91 | 0.08 | [0.78 – 1.06] | -1.20 |
| Old Adults Warmth | 0.89 | 0.13 | [0.69 – 1.14] | -0.91 |
| Old Adults Competence | 0.89 | 0.12 | [0.91 – 1.48] | 1.18 |
| Young Adults Warmth | 0.80 | 0.16 | [0.58 – 1.09] | -1.42 |
| Young Adults Competence | 1.31 | 0.15 | [0.98 – 1.76] | 1.81 |
| Internal Motivation to Respond w/o Prejudice | 1.17 | 0.09 | [0.98 – 1.39] | 1.70 |
| Child Age | 1.11 | 0.03 | [1.03 – 1.20] | 2.77** |

*Note*: ** p* < .05, ** *p* < .01, *** *p* < .001

**Table S13. Discussion likelihood as a function of beliefs and attitudes toward racial groups**

| **Predictors** | **OR** | **SE** | **95%CI** | ***z*** |
| --- | --- | --- | --- | --- |
| Racial Essentialism | 1.34 | 0.09 | [1.12 – 1.60] | 3.24** |
| Racial Social Constructionism | 1.24 | 0.11 | [1.01 – 1.53] | 2.01* |
| Racial Category Blindness | 0.91 | 0.10 | [0.76 – 1.10] | -0.94 |
| Black People Warmth | 1.65 | 0.18 | [1.16 – 2.34] | 2.77** |
| Black People Competence | 0.64 | 0.17 | [0.46 – 0.90] | -2.55* |
| White People Warmth | 0.98 | 0.17 | [0.70 – 1.35] | -0.14 |
| White People Competence | 0.91 | 0.16 | [0.66 – 1.25] | -0.58 |
| Internal Motivation to Respond w/o Prejudice | 1.12 | 0.11 | [0.91 – 1.39] | 1.06 |
| Child Age | 1.09 | 0.04 | [1.01 – 1.17] | 2.21* |

*Note*: ** p* < .05, ** *p* < .01, *** *p* < .001

**Table S14. Discussion likelihood as a function of beliefs and attitudes toward social class groups**

| **Predictors** | **OR** | **SE** | **95%CI** | ***z*** |
| --- | --- | --- | --- | --- |
| Social Class Essentialism | 1.54 | 0.10 | [1.27 – 1.86] | 4.35*** |
| Social Class Social Constructionism | 1.16 | 0.12 | [0.91 – 1.46] | 1.21 |
| Social Class Category Blindness | 0.90 | 0.08 | [0.76 – 1.05] | -1.34 |
| Poor People Warmth | 0.96 | 0.16 | [0.71 – 1.31] | -0.25 |
| Poor People Competence | 0.85 | 0.15 | [0.63 – 1.15] | -1.03 |
| Rich People Warmth | 1.12 | 0.11 | [0.91 – 1.37] | 1.07 |
| Rich People Competence | 0.98 | 0.11 | [0.78 – 1.22] | -0.22 |
| Internal Motivation to Respond w/o Prejudice | 1.11 | 0.10 | [0.90 – 1.36] | 0.96 |
| Child Age | 1.07 | 0.04 | [0.99 – 1.15] | 1.72 |

*Note*: ** p* < .05, ** *p* < .01, *** *p* < .001

**Table S15: Discussion likelihood as a function of explanation endorsement for the age comparison**

| **Predictors** | **OR** | **SE** | **95%CI** | ***z*** |
| --- | --- | --- | --- | --- |
| Behavioral [Yes] | 1.20 | 0.21 | [0.80 – 1.79] | 0.87 |
| Biological [Yes] | 2.44 | 0.22 | [1.60 – 3.71] | 4.15*** |
| Structural [Yes] | 1.29 | 0.22 | [0.83 – 1.98] | 1.14 |

*Note*: For each explanation, the estimate represents the OR of discussion likelihood changing when they endorsed (said yes) an explanation compared to participants who did not endorse (said no) an explanation. ** p* < .05, ** *p* < .01, *** *p* < .001.

**Table S16: Discussion likelihood as a function of explanation endorsement for the race comparison**

| **Predictors** | **OR** | **SE** | **95%CI** | ***z*** |
| --- | --- | --- | --- | --- |
| Behavioral [Yes] | 1.43 | 0.21 | [0.95 – 2.16] | 1.70 |
| Biological [Yes] | 1.16 | 0.18 | [0.81 – 1.67] | 0.82 |
| Structural [Yes] | 1.51 | 0.21 | [1.01 – 2.25] | 2.01* |

*Note*: For each explanation, the estimate represents the OR of discussion likelihood changing when they endorsed (said yes) an explanation compared to participants who did not endorse (said no) an explanation. ** p* < .05, ** *p* < .01, *** *p* < .001.

**Table S17: Discussion likelihood as a function of explanation endorsement for the social class comparison**

| **Predictors** | **OR** | **SE** | **95%CI** | ***z*** |
| --- | --- | --- | --- | --- |
| Behavioral [Yes] | 1.77 | 0.20 | [1.19 – 2.64] | 2.80** |
| Biological [Yes] | 1.16 | 0.18 | [0.82 – 1.65] | 0.84 |
| Structural [Yes] | 1.49 | 0.20 | [1.02 – 2.19] | 2.06* |

*Note*: For each explanation, the estimate represents the OR of discussion likelihood changing when they endorsed (said yes) an explanation compared to participants who did not endorse (said no) an explanation. ** p* < .05, ** *p* < .01, *** *p* < .001.

**Table S18a. Wald 𝜒2 test predicting if the presence of the ‘explanation’ code differed by disparity type**

| **Predictor** | **𝜒2** | **df** | ***p*** |
| --- | --- | --- | --- |
| Disparity Type | 128.45 | 2 | < .001 |

**Table S18b. Pairwise comparisons of ‘explanation’ code by disparity type**

| **Pairwise Comparisons** | **OR** | **SE** | **95%CI** | ***z*** |
| --- | --- | --- | --- | --- |
| Age – Social Class | 2.10 | 0.28 | [1.36, 3.22] | 4.03** |
| Age – Race | 9.83 | 2.04 | [6.05, 15.97] | 11.04*** |
| Social Class – Race | 4.69 | 0.87 | [3.04, 7.23] | 8.39*** |

*Note:* P-values were adjusted using Tukey’s method for comparing a family of 3 estimates within each comparison of code per disparity judgment. ** p* < .05, ** *p* < .01, *** *p* < .001.

**Table S19a. Wald 𝜒2 test predicting if the presence of the ‘biological’ code differed by disparity type**

| **Predictor** | **𝜒2** | **df** | ***p*** |
| --- | --- | --- | --- |
| Disparity Type | 136.09 | 2 | < .001 |

**Table S19b. Pairwise comparisons of ‘biological code’ by disparity type**

| **Pairwise Comparisons** | **OR** | **SE** | **95%CI** | ***z*** |
| --- | --- | --- | --- | --- |
| Age – Social Class | 250.53 | 0.49 | [79.63, 788.12] | 11.3*** |
| Age – Race | 51.82 | 0.38 | [21.39, 125.52] | 10.46*** |
| Social Class – Race | 0.21 | 0.32 | [0.09, 0.44] | -4.93*** |

*Note:* P-values were adjusted using Tukey’s method for comparing a family of 3 estimates within each comparison of code per disparity judgment. ** p* < .05, ** *p* < .01, *** *p* < .001.

**Table S20a. Wald 𝜒2 test predicting if the presence of the ‘behavioral’ code differed by disparity type**

| **Predictor** | **𝜒2** | **df** | ***p*** |
| --- | --- | --- | --- |
| Disparity Type | 22.25 | 2 | < .001 |

**Table S20b. Pairwise comparisons of ‘behavioral’ code by disparity type**

| **Pairwise Comparisons** | **OR** | **SE** | **95%CI** | ***z*** |
| --- | --- | --- | --- | --- |
| Age – Social Class | 0.03 | 0.02 | [0.01, 0.18] | -4.54*** |
| Age – Race | 0.03 | 0.02 | [0.01, 0.18] | -4.53*** |
| Social Class – Race | 1.00 | 0.43 | [0.36, 2.76] | 1.00 |

*Note:* P-values were adjusted using Tukey’s method for comparing a family of 3 estimates within each comparison of code per disparity judgment. ** p* < .05, ** *p* < .01, *** *p* < .001.

**Table S21a. Wald 𝜒2 test predicting if the presence of the ‘structural’ code differed by disparity type**

| **Predictor** | **𝜒2** | **df** | ***p*** |
| --- | --- | --- | --- |
| Disparity Type | 118.12 | 2 | < .001 |

**Table S21b. Pairwise comparisons of ‘structural’ code by disparity type**

| **Pairwise Comparisons** | **OR** | **SE** | **95%CI** | ***z*** |
| --- | --- | --- | --- | --- |
| Age – Social Class | 0.01 | 0.01 | [0.004, 0.01] | -7.96** |
| Age – Race | 0.03 | 0.02 | [0.004, 0.14] | -4.99*** |
| Social Class – Race | 11.03 | 2.84 | [6.04, 20.16] | 9.33*** |

*Note:* P-values were adjusted using Tukey’s method for comparing a family of 3 estimates within each comparison of code per disparity judgment. ** p* < .05, ** *p* < .01, *** *p* < .001.

**Table S22a. Wald 𝜒2 test predicting if the presence of the ‘everyone is equal’ code differed by disparity type**

| **Predictor** | **𝜒2** | **df** | ***p*** |
| --- | --- | --- | --- |
| Disparity Type | 33.01 | 2 | < .001 |

**Table S22b. Pairwise comparisons of ‘everyone is equal’ code by disparity type**

| **Pairwise Comparisons** | **OR** | **SE** | **95%CI** | ***z*** |
| --- | --- | --- | --- | --- |
| Age – Social Class | 0.12 | 0.07 | [0.03, 0.44] | -3.86*** |
| Age – Race | 0.04 | 0.02 | [0.01, 0.14] | -5.74*** |
| Social Class – Race | 0.30 | 0.13 | [0.11, 0.83] | -2.77* |

*Note:* P-values were adjusted using Tukey’s method for comparing a family of 3 estimates within each comparison of code per disparity judgment. ** p* < .05, ** *p* < .01, *** *p* < .001.

**Table S23a. Wald 𝜒2 test predicting if the presence of the ‘preventative’ code differed by disparity type**

| **Predictor** | **𝜒2** | **df** | ***p*** |
| --- | --- | --- | --- |
| Disparity Type | 10.54 | 2 | .005 |

**Table S23b. Pairwise comparisons of ‘preventative’ code by disparity type**

| **Pairwise Comparisons** | **OR** | **SE** | **95%CI** | ***z*** |
| --- | --- | --- | --- | --- |
| Age – Social Class | 1.78 | 0.39 | [1.06, 2.99] | 2.26* |
| Age – Race | 1.90 | 0.42 | [1.13, 3.19] | 2.87* |
| Social Class – Race | 1.06 | 0.25 | [0.61, 1.84] | 0.26 |

*Note:* P-values were adjusted using Tukey’s method for comparing a family of 3 estimates within each comparison of code per disparity judgment. ** p* < .05, ** *p* < .01, *** *p* < .001.

**Table S24. Wald 𝜒2 test predicting if the presence of the ‘reassurance’ code differed by disparity type**

| **Predictor** | **𝜒2** | **df** | ***p*** |
| --- | --- | --- | --- |
| Disparity Type | 0.14 | 2 | .93 |

**Table S25a. Wald 𝜒2 test predicting if the presence of the ‘deflection’ code differed by disparity type**

| **Predictor** | **𝜒2** | **df** | ***p*** |
| --- | --- | --- | --- |
| Disparity Type | 7.51 | 2 | .02 |

**Table S25b. Pairwise comparisons of ‘deflection’ code by disparity type**

| **Pairwise Comparisons** | **OR** | **SE** | **95%CI** | ***z*** |
| --- | --- | --- | --- | --- |
| Age – Social Class | 0.42 | 0.40 | [0.04, 3.82] | -0.91 |
| Age – Race | 0.07 | 0.07 | [0.01, 0.72] | -2.67* |
| Social Class – Race | 0.16 | 0.15 | [0.02, 1.45] | -1.95 |

*Note:* P-values were adjusted using Tukey’s method for comparing a family of 3 estimates within each comparison of code per disparity judgment. ** p* < .05, ** *p* < .01, *** *p* < .001.

**Table S26a. Wald 𝜒2 test predicting if the presence of the ‘would not answer’ code differed by disparity type**

| **Predictor** | **𝜒2** | **df** | ***p*** |
| --- | --- | --- | --- |
| Disparity Type | 24.42 | 2 | < .001 |

**Table S26b. Pairwise comparisons of ‘would not answer’ code by disparity type**

| **Pairwise Comparisons** | **OR** | | **SE** | | **95%CI** | | ***z*** | |
| --- | --- | --- | --- | --- | --- | --- | --- | --- |
| Age – Social Class | 0.36 | 0.13 | | [0.15, 0.85] | | -2.77* | |  |
| Age – Race | 0.16 | 0.06 | | [0.06, 0.38] | | -4.93*** | |  |
| Social Class – Race | 0.43 | 0.14 | | [0.20, 0.93] | | -2.56* | |  |

*Note:* P-values were adjusted using Tukey’s method for comparing a family of 3 estimates within each comparison of code per disparity judgment. ** p* < .05, ** *p* < .01, *** *p* < .001.

**Table S27a. Wald 𝜒2 test predicting if the presence of the ‘rejection of information’ code differed by disparity type**

| **Predictor** | **𝜒2** | **df** | ***p*** |
| --- | --- | --- | --- |
| Disparity Type | 47.01 | 2 | < .001 |

**Table S27b. Pairwise comparisons of ‘rejection of information’ code by disparity type**

| **Pairwise Comparisons** | **OR** | **SE** | **95%CI** | ***z*** |
| --- | --- | --- | --- | --- |
| Age – Social Class | 0.19 | 0.08 | [0.07, 0.53] | -3.80*** |
| Age – Race | 0.04 | 0.02 | [0.01, 0.11] | -6.80*** |
| Social Class – Race | 0.20 | 0.08 | [0.08, 0.49] | -4.20*** |

*Note:* P-values were adjusted using Tukey’s method for comparing a family of 3 estimates within each comparison of code per disparity judgment. ** p* < .05, ** *p* < .01, *** *p* < .001.

**Table S28a. Wald 𝜒2 test predicting if the presence of the ‘search for information’ code differed by disparity type**

| **Predictor** | **𝜒2** | **df** | ***p*** |
| --- | --- | --- | --- |
| Disparity Type | 13.81 | 2 | .001 |

**Table S28b. Pairwise comparisons of ‘search for information’ code by disparity type**

| **Pairwise Comparisons** | **OR** | **SE** | | **95%CI** | | ***z*** | |
| --- | --- | --- | --- | --- | --- | --- | --- |
| Age – Social Class | 0.45 | 0.41 | [0.07, 0.53] | | -0.87 | |  |
| Age – Race | 0.01 | 0.01 | [0.01, 0.11] | | -3.64*** | |  |
| Social Class – Race | 0.03 | 0.03 | [0.08, 0.49] | | -3.14** | |  |

*Note:* P-values were adjusted using Tukey’s method for comparing a family of 3 estimates within each comparison of code per disparity judgment. ** p* < .05, ** *p* < .01, *** *p* < .001.

**Table S29a. Wald 𝜒2 test predicting if the presence of the ‘expressing uncertainty’ code differed by disparity type**

| **Predictor** | **𝜒2** | **df** | ***p*** |
| --- | --- | --- | --- |
| Disparity Type | 55.14 | 2 | < .001 |

**Table S29b. Pairwise comparisons of ‘expressing uncertainty’ code by disparity type**

| **Pairwise Comparisons** | **OR** | **SE** | **95%CI** | ***z*** | |
| --- | --- | --- | --- | --- | --- |
| Age – Social Class | 0.39 | 0.11 | [0.20, 0.77] | -3.23** |  |
| Age – Race | 0.09 | 0.03 | [0.04, 0.20] | -7.16*** |  |
| Social Class – Race | 0.23 | 0.06 | [0.13, 0.43] | -5.56*** |  |

*Note:* P-values were adjusted using Tukey’s method for comparing a family of 3 estimates within each comparison of code per disparity judgment. ** p* < .05, ** *p* < .01, *** *p* < .001.

**Table S30a. Wald 𝜒2 test predicting if the presence of the ‘important’ code differed by disparity type**

| **Predictor** | **𝜒2** | **df** | ***p*** |
| --- | --- | --- | --- |
| Disparity Type | 13.37 | 2 | .001 |

**Table S30b. Pairwise comparisons of ‘important’ code by disparity type**

| **Pairwise Comparisons** | **OR** | **SE** | **95%CI** | ***z*** |
| --- | --- | --- | --- | --- |
| Age – Social Class | 1.55 | 0.32 | [0.95, 2.52] | 2.12 |
| Age – Race | 2.20 | 0.48 | [1.32, 3.67] | 3.62*** |
| Social Class – Race | 1.42 | 0.32 | [0.84, 2.39] | 1.57 |

*Note:* P-values were adjusted using Tukey’s method for comparing a family of 3 estimates within each comparison of code per disparity judgment. ** p* < .05, ** *p* < .01, *** *p* < .001.

**Table S31. Wald 𝜒2 test predicting if the presence of the ‘prompted only’ code differed by disparity type**

| **Predictor** | **𝜒2** | **df** | ***p*** |
| --- | --- | --- | --- |
| Disparity Type | 6.05 | 2 | .05 |

**Table S32a. Wald 𝜒2 test predicting if the presence of the ‘shielding’ code differed by disparity type**

| **Predictor** | **𝜒2** | **df** | ***p*** |
| --- | --- | --- | --- |
| Disparity Type | 8.96 | 2 | .01 |

**Table S32b. Pairwise comparisons of ‘shielding’ code by disparity type**

| **Pairwise Comparisons** | **OR** | **SE** | **95%CI** | ***z*** |
| --- | --- | --- | --- | --- |
| Age – Social Class | 0.39 | 0.13 | [0.18, 0.83] | -2.92** |
| Age – Race | 0.49 | 0.16 | [0.23, 1.04] | -2.22 |
| Social Class – Race | 1.26 | 0.38 | [0.62, 2.55] | 0.75 |

*Note:* P-values were adjusted using Tukey’s method for comparing a family of 3 estimates within each comparison of code per disparity judgment. ** p* < .05, ** *p* < .01, *** *p* < .001.

**Table S33. *M(SD)* and bivariate correlations of individual difference variables predicting parents’ likelihood of discussing the age disparity**

|  | *M (SD)* | 1 | 2 | 3 | 4 | 5 | 6 | 7 | 8 | 9 |
| --- | --- | --- | --- | --- | --- | --- | --- | --- | --- | --- |
| 1. Social Constructionism | 4.96 (1.13) | - |  |  |  |  |  |  |  |  |
| 2. Essentialism | 4.82 (1.15) | **.68** | - |  |  |  |  |  |  |  |
| 3. Category Blindness | 5.24 (1.30) | **.35** | **.22** | - |  |  |  |  |  |  |
| 4. Old Warmth | 3.94 (0.94) | **.30** | **.37** | **.26** | - |  |  |  |  |  |
| 5. Old Competence | 3.51 (1.06) | **.36** | **.36** | **.24** | **.61** | - |  |  |  |  |
| 6. Young Warmth | 3.39 (1.05) | **.39** | **.34** | **.18** | **.34** | **.57** | - |  |  |  |
| 7. Young Competence | 3.47 (1.08) | **.38** | **.32** | **.19** | **.40** | **.52** | **.82** | - |  |  |
| 8. IMS | 5.38 (1.12) | **.20** | .07 | **.36** | **.24** | .01 | .02 | .04 | - |  |
| 9. Child’s Age | 8.59  (2.28) | .09 | .08 | .09 | .08 | .06 | .07 | .05 | .01 | - |

**Note:** Values in bold represent significant correlations, p < .05.

**Table S34. *M(SD)* and bivariate correlations of individual difference variables predicting parents’ likelihood of discussing the racial disparity**

|  | *M (SD)* | 1 | 2 | 3 | 4 | 5 | 6 | 7 | 8 | 9 |
| --- | --- | --- | --- | --- | --- | --- | --- | --- | --- | --- |
| 1. Social Constructionism | 5.01 (1.14) | - |  |  |  |  |  |  |  |  |
| 2. Essentialism | 4.45 (1.31) | **.55** | - |  |  |  |  |  |  |  |
| 3. Category Blindness | 5.56 (1.31) | **.36** | .06 | - |  |  |  |  |  |  |
| 4. Black Warmth | 3.46 (1.10) | **.28** | **.36** | **.21** | - |  |  |  |  |  |
| 5. Black Competence | 3.55 (1.10) | **.28** | **.34** | **.22** | **.87** | - |  |  |  |  |
| 6. White Warmth | 3.61 (0.99) | **.32** | .**30** | **.27** | **.49** | **.43** | - |  |  |  |
| 7. White Competence | 3.73 (0.96) | **.34** | **.24** | **.32** | **.33** | **.36** | **.78** | - |  |  |
| 8. IMS | 5.38 (1.12) | **.28** | **-.14** | **.60** | .01 | .04 | **.14** | **.25** | - |  |
| 9. Child’s Age | 8.59  (2.28) | **.08** | **.13** | .01 | .07 | .10 | .05 | .03 | .01 | - |

**Note:** Values in bold represent significant correlations, p < .05.

**Table S35. *M(SD)* and bivariate correlations of individual difference variables predicting parents’ likelihood of discussing the social class disparity**

|  | *M (SD)* | 1 | 2 | 3 | 4 | 5 | 6 | 7 | 8 | 9 |
| --- | --- | --- | --- | --- | --- | --- | --- | --- | --- | --- |
| 1. Social Constructionism | 5.03 (1.07) | - |  |  |  |  |  |  |  |  |
| 2. Essentialism | 4.60 (1.22) | **.63** | - |  |  |  |  |  |  |  |
| 3. Category Blindness | 5.46 (1.35) | **.35** | **.09** | - |  |  |  |  |  |  |
| 4. Poor Warmth | 3.39 (1.14) | **.26** | **.33** | **.16** | - |  |  |  |  |  |
| 5. Poor Competence | 3.29 (1.20) | **.21** | **.34** | .08 | **.85** | - |  |  |  |  |
| 6. Rich Warmth | 3.16 (1.20) | **.28** | **.27** | .08 | **.41** | **.46** | - |  |  |  |
| 7. Rich Competence | 3.73 (1.06) | **.33** | **.17** | **.21** | **.30** | **.26** | **.60** | - |  |  |
| 8. IMS | 5.38 (1.12) | **.28** | -.04 | **.53** | .00 | -.04 | -.06 | **.20** | - |  |
| 9. Child’s Age | 8.59  (2.28) | .05 | .08 | .04 | .09 | .08 | .04 | .02 | .01 | - |

**Note:** Values in bold represent significant correlations, p < .05.
