# Supplementary material for: Who gets sicker and why? Parents’ perceptions of COVID-19 disparities and how they would explain them to their children
Source: PLoS One. 2025 Oct 15;20(10):e0332140. doi: 10.1371/journal.pone.0332140 (PMC12527206; doi:10.1371/journal.pone.0332140)
Supplement: Attachment — Submitted filename: MejiaGomezEtAl_RebuttalLetter_4.16.25.docx [file pone.0332140.s003.docx]

PONE-D-24-52344

**EDITOR’S COMMENTS**

1. No information about the methods was provided in the abstract. Please provide information on the types of quantitative and qualitative data analysis used.

We have expanded the abstract to include this information.

2. Please add information about the demographics of the participants to the abstract.

This has been added as well.

3. What are your findings of each analysis? Please provide clear and concise information in the abstract.

These are now included.

4. Please avoid any promotional statements in the manuscript. (e.g., "No research has looked at.."). It is not sufficient to indicate that a research topic has not been examined before in a way that the authors will examine it.

Although we agree that this is not sufficient motivation, we do feel that it’s relevant in terms of understanding the context for this work. We have reframed such statements to be informative rather than promotional.

5. The current study section should briefly describe the research gap, aims, hypotheses/ research questions (RQs).

We have restructured the Current Study section to include this information.

6. The rationales should be provided in the literature review, not in the current study section. Avoid any new information or new citations in this section.

Thank you for this suggestion. We have moved these rationales to the literature review, and then briefly note them as relevant to the aims in the Current Study section.

7. What are your directional hypotheses?  Please explain them in the "current study" section.

We originally mentioned this information in the Method section, but now clearly state that we did not have directional hypotheses in the Current Study section.

8. Which analysis was used for each hypothesis/ RQs? Justification is needed for each analysis (e.g., mixed effect logistic regression). Please see reviewers' comments.

We now explicitly state our research questions in the Current Study section and now organize the Results in terms of research questions with their corresponding analyses.

9. The current version of the results section is poorly structured and difficult for readers to follow. Readers cannot manage that much information. I recommend providing a brief reminder to readers of which analysis was used for each aim and/or hypothesis.

As stated in response to point #8 above, we have restructured the results in line with the Editor’s suggestion.

10. What are your most important novel findings? Please explain in the first paragraph of the discussion.

Thank you for this helpful suggestion. We have explained the most important novel findings in the first paragraph of the discussion.

**REVIEWER 1**

1. Thank you for allowing me to review this paper. The problem is well developed (albeit lengthily--but given the complexity of the research question and its theoretical underpinnings and merits, this is acceptable). The analytical decisions are well explained. Every strand of the findings are thoroughly discussed.

Thank you for the kind comments.

2. I only have one recommendation, and that is to outline multisectoral interventions/policy implications that can be derived from the study.

Although we are not familiar with multisectoral interventions, we have added a section on the implications of our work (see also responses to Reviewer 2). If the reviewer would still like us to add information on these types of interventions, we would appreciate a citation or further guidance.

**REVIEWER 2**

1. Acknowledgement of disparities in health is important, and this topic has potential to make a contribution to the research in this area.

Thank you for this comment.

2. However, as this paper has a strong focus relating to the covid-19 pandemic, the links to general discussion/acknowledgement of health disparities and the potential impact of this work is not clear.

We have strengthened these portions in the General Discussion (see also Reviewer 1’s suggestion about expanding on the policy implications).

3. It would be useful to strengthen the rationale for this work – what are the wider implications of this study and its results? The relationships between parental beliefs and discussion of health disparities with young children may differ based on the type of virus (as acknowledged in some of the parent responses, their conversations may have changed due to specific concerns/risks related specifically to covid-19) and this should be acknowledged/discussed in the paper.

We now expand on the rationale and wider implications of the work, as well as the potential for different results based on the type of virus (in the Discussion).

4. The methods of this paper should be expanded. Much of this section includes results and instead should describe the questionnaire development, distribution, recruitment, etc, along with a description of the analysis plan. Could the authors expand on the process for modifying existing tools/questionnaires for this questionnaire – were they pilot tested or validated for use with this study population?

We have added brief descriptions to expand on these points. We added that the disparity judgment and explanation endorsement tasks were derived from Menendez et al. (2024). We explained that the disparity judgment scale task was added to make a potentially more sensitive measure. Minor wording modifications are not uncommon in survey research, so we took discretion to alter the wording to apply each construct to the relevant social category and group. All of the individual difference measures were validated scales and were found to be reliable in the current study, according to the observed alphas (all αs > .75). Also, the Materials and method section and the Results section are separated.

5. In the results, several models were included and described but this was difficult to follow. It would be useful to have some descriptive statistics (e.g. baseline characteristics of the study population, response rate (if known), distribution of the response options to questions, proportion of missing responses, etc). This might help justify/explain the choice for various regression models for the reader. For the qualitative coding, why were these results included in a regression model instead of processed as qualitative data?

We have added a table with the descriptives for the individual difference measures, as requested. We also now note that, because we observed that the codes appeared to differ by category (age, class, race), we conducted pre-registered regression models in order to test whether these were stable differences in our sample.

6. The discussion provided a good general overview of results, but it would be useful to review the literature on parental beliefs regarding disparities and how that impacts discussions with children, and how the results from this study may compare. As mentioned above, I believe further reflection on the implications of this work is needed.

We expand on these issues in the implications section of the ms. We believe this work could inform possible interventions to help parents understand how to discuss these topics with their children.

**Below is our Amended Funding Statement, as requested:**

This research was supported by NSF Grant #2055164 to SAG. Any opinions, findings, and conclusions and recommendations expressed in this material are those of the authors and do not necessarily reflect the view of the National Science Foundation. There was no additional external funding received for this study. The funders had no role in study design, data collection and analysis, decision to publish, or preparation of the manuscript.
