# Supplementary material for: Who gets sicker and why? Parents’ perceptions of COVID-19 disparities and how they would explain them to their children
Source: PLoS One. 2025 Oct 15;20(10):e0332140. doi: 10.1371/journal.pone.0332140 (PMC12527206; doi:10.1371/journal.pone.0332140)
Supplement: Attachment — Submitted filename: Response to Reviewers.docx [file pone.0332140.s004.docx]

PONE-D-24-52344R1

**Reviewer 3**

This is a complex, multi-faceted study that took on the enormous yet worthy task of examining how parents think about health disparities across multiple social categories and how their beliefs and motivations influence what they might discuss with their children. It appears the authors have made substantial efforts to revise the manuscript after the initial reviews. Upon my reading, a strong rationale is provided, research questions for an exploratory study are clearly outlined, and the methods are well-described. Organizing results by research question was extremely helpful to maintain focus even while addressing findings across different categories. Overall, I recommend this article for publication with minor revisions. Below I make recommendations to clarify a few points and to expand on discussion of the findings.

Thank you for the positive comments.

Introduction: The authors provide sufficient (but not overwhelming) background information to ensure the reader has adequate context for the study design and analysis. There is an effort to equally attend to health disparities and parent socialization in relation to multiple social categories (age, race, social class) of relevance, despite disparate and often sparse bodies of literature.

1. One minor point of confusion was in the description of relevant constructs (p.8-10); it was stated that 5 constructs would be discussed, but only 4 subheadings are presented. It would help to include in the final sub-section a clarifying statement that essentialism and social constructionism are the fourth and fifth construct and explain why they are being discussed together (or to separate social constructionism into its own subheading).

Thank you for your suggestion. We have separated essentialism and social constructionism by subheading to clarify that they are conceptually distinct constructs.

Methods:

1. Under participants, given that all other demographics are listed as “self-reported,” the final statement that the sample was neither conservative nor liberal reads as an objective assessment; recommend clarifying that this is a statement of the average self-rating of participants. Conversely, it may be more concise to state early in this section that all demographic characteristics were self-reported.

We added a brief statement indicating that all demographics were self-reported.

2. Clarify for IMS whether there were 5 items for each of the 3 social categories (age, race, and social class) or only 5 items total. If only 5 total, creating 1 scale, why was this measure collapsed across groups whereas other measures had separate scales for each social category?

The measure was 5 items total, and we have clarified this in the Method section. The reason we did not have separate questions for each social category was that we did not want to presuppose which category (out of the pair) participants were more prejudiced about. For example, some participants may have more prejudice against rich people than poor people, and others may have had the reverse. None of the other scales that we had involved picking just one of the two categories in a pair. What we most cared about was whether participants were motivated not to show prejudice broadly; therefore, the single 5-item scale was slightly modified to include general wording.

3. There remains debate about the appropriate kappa level to be deemed “reliable.” (p.19, line 387). Assessing that codes with kappa > .60 had acceptable interrater reliability would more appropriately be described as having moderate to strong/substantial agreement (e.g. McHugh, 2012; Hallgren, 2012).

We now acknowledge this in our qualitative coding section.

Results:

1. Inconsistency in how measures are referred to and capitalized between RQs (e.g. “Disparity Judgment” p.22 and p. 26 vs “disparity judgment (forced-choice) task” p. 24). If these analyses use the same data, then would be best to clarify in RQ1 that the force-choice task data was used; if not, note it was the scale.

We apologize for the inconsistencies, which have been corrected.

2. The indication of significance levels in Table 5 is extremely confusing given the text explains only specific comparisons met the corrected alpha level. Recommend providing all p-values in a separate column and using an indicator (perhaps bold font as in Table 4) to show only those that were significant at p> .017.

We have clarified that all selections for the disparity judgment (forced-choice) task were significantly different from chance (33.33%), noting that some were below chance and others were above chance. We also indicate that all comparisons to chance were p < .001.

3. Similarly to previous comment, clarify on p. 25 line 494 that this analysis now uses the disparity judgment scale task. This allows readers to refer back to the Methods if desired to remind themselves how this measure was administered.

We have briefly added that these analyses were for the disparity judgment (scale) task.

Discussion:

1. The example provided on p.32, line 615 about protecting children from emotional harm regarding age and a grandparent seems inconsistent with the sentence and citations that follow which focus on parents’ unwillingness to discuss racial topics and that young children are aware of social inequalities. This example may fit better elsewhere.

We appreciate the suggestion, and have reorganized the paragraphs in this section so that the examples and citations are more appropriate for the points that are made.

2. The final sentence on p.33-34 does not need to be its own paragraph. Append it to the previous one or split this section into paragraphs in a logical place (perhaps line 650, “Parents’ explanation…”)

We have added that final sentence to the prior paragraph.

3. Some findings are summarized but not discussed. Why do the authors believe certain differences were found in light of previous literature? What are the implications of such findings?

We have added to the Discussion, adding interpretations and implications for findings that had been only summarized.

- Why were behavioral explanations more likely when discussing social class disparities(p.34)? Knowledge about healthcare access is mentioned, but this seems to be a structural explanation. How could this relate to behavior?

To clarify: it is not that behavioral explanations were more likely for social class disparities (rather, as seen in Table 8, structural explanations were more likely). Instead, the finding discussed on p. 34 was that parents who provided behavioral explanations were more likely to say they would discuss these disparities with their children. We suspect that the reviewer is correct, that behavioral explanations may often be assumed to be the result of structural factors. Importantly, as noted, parents who provided structural explanations were also more likely to say they would discuss these disparities with their children. We have edited this paragraph to make these findings clearer.

- Similarly, why is the finding regarding political affiliation meaningful (p.34)? More discussion is needed regarding how this relates to past research, parent socialization practices, and/or implications for children’s development of awareness of health disparities.

We have elaborated on this finding and how it relates to past research.

- Further explanation for the social class difference related to essentialism is necessary (p.35). This seems consistent with prior research on social class beliefs in the US and that being poor is related to character rather than structural issues.

We have expanded our interpretation regarding this finding in the Discussion section.

4. I appreciate the effort of the authors to address implications more thoroughly. However, it seems an acknowledgement should be made that some parents may be uninterested in learning about the existence of health disparities and that this study demonstrates that even providing established statistics did not change some parents’ minds about whether the disparity existed. The idea of an intervention focused on helping parents understand the value of “accurate explanatory frameworks” neglects the current political and social climate where what is viewed as “accurate” and factual seems up for debate. I wonder whether the authors have any suggestions for how to leverage parents’ understanding that some health disparities exist (e.g. for age) to create openness to recognizing others.

We have added an acknowledgement that our results may indicate parents’ disinterest in learning about social disparities in the Discussion. We now also acknowledge the role of the politicalization of COVID-19 policies and health recommendations, and suggest that in future research it would be of interest to determine how parents reason about other health disparities that may not be subject to the same level of politicalization as COVID-19 (e.g., the flu). We seriously considered the reviewer’s interesting suggestion of leveraging parents’ understanding of (e.g.) age disparities, as a foundation for introducing other disparities. In the end, however, we opted not to add this suggestion to the ms., as we are concerned that doing so could encourage parents to treat other disparities (e.g., race, social class) as biological, much as they do for age.

5. The paragraph on policy focuses more generally on adults and healthcare workers, but needs to find a way to circle back to parents and children. Given that parents may not be aware of (or accepting of) health disparities, an alternate way to approach this would be to consider what role other sources of socialization (educator, peers, health professionals, media) have on the development of children’s beliefs in addition to parents, particularly as children age.

We have incorporated some discussion on potential ways that other socialization agents (i.e., educators, health professionals) can contribute to children’s developing beliefs of health disparities.

**Reviewer 4**

1. Abstract: authors begin by discussing COVID health disparities and then jump to posing the question of whether parents discuss these disparities with their children. There seems to be a link missing. Perhaps open the abstract with the importance of promoting parent-child conversations about health disparities (such as covid). Authors also mention that they employed a “series of individual difference measures”. I think authors need to be a bit more specific so that the reader knows what types of measures were employed (without having to list them all).

We made these edits.

2. I was surprised to see that the paragraphs about the importance of parent-child conversations were placed so late into the introduction. I suggest moving them earlier and elaborating upon how these conversations shaped child outcomes (there’s research showing links between conversational content and youth outcomes during the pandemic).

We have adjusted parts of the Introduction and added recent research findings emphasizing the role of parent-child conversations on children’s social and conceptual development in the COVID-19 context.

3. The connections between authors’ first aims and the aim regarding individual differences in attitudes weren’t well streamlined. The attitudes/beliefs authors explore aren’t integrated within a theoretical framework, which makes it difficult to understand why authors selected all of these (i.e., how are these attitudes and beliefs differentiated and how do they conceptually relate to one another? How does it meaningfully contribute to authors’ overarching narrative). I urge authors to think about which attitudes/beliefs are core to their investigation and perhaps remove or move analyses concerning others to the supplementary material.

Given that these measures were pre-registered, we do not feel comfortable excluding them from the manuscript. However, we note that we took an exploratory approach, given little prior work addressing these questions. We also added our rationale for selecting these measures, namely, they have been found to relate to how people think about social categories and/or parenting behaviors associated with conversations about social categories.

4. I was surprised that authors did not have any specific hypotheses, especially for aim 4 as there is some research on this topic already (at least in the domain of race-based conversations)

Given the exploratory nature of Research Question 4, we did not preregister any hypotheses. For this reason, we do not add any post-hoc hypotheses for this research question.

5. Authors reported a sensitivity analysis in g*power but it sounds like they conducted an a priori power analysis to determine their sample size. Authors are also missing information about total predictors, tested predictors, power value, and alpha value.

The reviewer is correct that we did an a priori power analysis, and we have made changes to the text to reflect this. We have also added the missing pieces of information that the reviewer requested to the manuscript.

6. Do authors know the States from which this data was primarily collected? It’s great that authors collected data on political affiliation—it definitely matters for what parents talk about. I recommend including political leaning as a covariate in all multivariate analyses, as well as parent and/or child gender and age (if not already a predictor)

We had at least one participant from each of 46 states and the District of Columbia, with the top three most common states being New York (n=36), Texas (n=36), and Florida (n=32). The states not represented in our data are Hawaii, Montana, Vermont, and Wyoming. We mention this in the Method. Regarding the covariates mentioned, we preregistered having the participants’ child’s age in our discussion likelihood models. We did not ask participants to indicate the gender of their child. Finally, we did not preregister including political leaning in our models. However, we conducted exploratory analyses predicting parents’ likelihood of discussing the disparities based on their highest educational attainment, political leaning, and political affiliation in three separate regression models, one for each disparity. We did not find political leaning to be a significant factor for discussing any of the three disparities, even when controlling for the socio-psychological individual differences.

7. Why didn’t authors explore race-related differences in their outcomes? 39% of the sample was bipoc—there may be some interesting differences in authors’ findings, especially the thematic analysis of content, strategies, and reasons parents provided.

The reviewer is correct that our sample consisted of a relatively large proportion of non-White participants. However, because our study’s design focused on Black-White differences, we do not believe it would be conceptually appropriate to collapse over all non-White participants, as we did not include information about disparities between other ethnic-racial groups. In our Limitations section, we advocate for future research to include more diverse samples to obtain perspectives of more individuals from groups who were most affected. Additionally, our data will be publicly available for anyone interested in conducting these analyses.

8. The parent explanation section is very interesting and could stand alone as a separate paper if authors were to further describe the findings qualitatively. Authors could unpack parents’ explanations further, especially regarding differences in themes by key demographics to determine who is saying what and how they are discussing the content. This would have important intervention implications.

Thank you for this suggestion. For the present paper, we will keep the parent explanation section succinct, but your idea is an interesting one that we can consider further in the future.

9. More information about the coding process is needed (i.e., how codes were developed and revised). Authors mention basing their coding scheme on Menendez et al, but it’s not clear whether the codebook was adapted for use with this data. Employing appropriate thematic analysis reporting procedures is recommended (Terry & Hayfield, 2021 could be helpful)

We first want to clarify that we did not employ a full thematic analysis, but thank the reviewer for guiding us on how to appropriately report our qualitative analyses. We employed a codebook approach with a combination of inductive and deductive codes. We have added information in the Method section about the process of creating the coding scheme, and provide details on which codes came from prior codebooks established in the literature and which were created specifically for this paper.

10. I’m unclear about what the purpose of the confidence ratings was.

The confidence ratings were included to include a more nuanced assessment, given that the disparity judgment was forced-choice and thus a fairly coarse measure. This point has been added to the manuscript.

11. RQ4: authors need to provide bivariate correlations between their attitudes/beliefs measures. The findings are quite perplexing. The results might be easier to digest if presented in a table rather than in text. The descriptives could be moved to the supplementary material. Was multicollinearity a factor if all variables were included in the same models?

We created a new table to display our Q4 results in a more digestible format. We added the descriptives and bivariate correlations to the Supporting Material. Additionally, multicollinearity was not an issue for any of the models, with all variance inflation factors below 5.05.

12. The political affiliation findings/discussion seem out of place.

We have adjusted the section where these findings are discussed, and expanded our interpretations of the political affiliation results to have a more cohesive discussion.

13. By the end of the paper, I am left wondering about the novelty of the findings. Authors present many results, but I think they could work toward crafting a more cohesive narrative to tie all the findings together. I think authors can leverage their findings in the implications section better.

We provided a brief overview of our findings in the abstract and added a conclusion section to provide a more cohesive narrative. Additionally, we have slightly restructured the implications section to better communicate how our findings can guide future research, intervention, and public policy.

14. There are a few typos throughout.

Thank you for alerting us to these. We have carefully gone through and hope we have corrected them all.
